# Supplementary material for: MetaRibo-Seq measures translation in microbiomes
Source: Nat Commun. 2020 Jun 29;11:3268. doi: 10.1038/s41467-020-17081-z (PMC7324362; doi:10.1038/s41467-020-17081-z)
Supplement: Supplementary file 10 — Supplementary Data 7 [file 41467_2020_17081_MOESM10_ESM.zip › File2/Confidence_High_Taxonomy/275963_out.krona.html]

Javascript must be enabled to view this page.

members
magnitude
magnitudeUnassigned
count
unassigned
taxon
rank

275963\_out

391

391
2
superkingdom

1239
2
391
phylum

SRS013800\_contig\_number\_contig-100\_36397.69689SRS1041143\_contig\_number\_contig-100\_9513.39305

389
186801
class

42
389
186802

SRS013951\_contig\_number\_31229SRS013965\_contig\_number\_42334SRS014287\_contig\_number\_20500SRS015217\_contig\_number\_25206SRS015794\_contig\_number\_contig-100\_399.94482SRS016381\_contig\_number\_contig-100\_13078.13079SRS016989\_contig\_number\_contig-100\_1419.102763SRS017191\_contig\_number\_11802SRS017307\_contig\_number\_13235SRS017821\_contig\_number\_33898SRS018888\_contig\_number\_contig-100\_18052.18053SRS019910\_contig\_number\_contig-100\_420.140694SRS021219\_contig\_number\_9343SRS022137\_contig\_number\_20243SRS024331\_contig\_number\_38887SRS047044\_contig\_number\_19511SRS054059\_contig\_number\_contig-100\_13987.13988SRS054956\_contig\_number\_15571SRS056537\_contig\_number\_contig-100\_3926.317846SRS062427\_contig\_number\_contig-100\_1195.78594SRS063518\_contig\_number\_34895SRS063985\_contig\_number\_31806SRS063985\_contig\_number\_contig-100\_2089.176693SRS065504\_contig\_number\_40178SRS077127\_contig\_number\_17875SRS077194\_contig\_number\_31092SRS077502\_contig\_number\_7811SRS078260\_contig\_number\_contig-100\_7629.7629SRS098571\_contig\_number\_contig-100\_1643.139696SRS098620\_contig\_number\_contig-100\_3670.3670SRS100907\_contig\_number\_contig-100\_36644.36645SRS104036\_contig\_number\_contig-100\_33682.33683SRS104311\_contig\_number\_34997SRS104693\_contig\_number\_contig-100\_2676.87427SRS140513\_contig\_number\_16064SRS142503\_contig\_number\_contig-100\_5553.269155SRS142890\_contig\_number\_43094SRS143417\_contig\_number\_34155SRS143991\_contig\_number\_contig-100\_5044.174096SRS146812\_contig\_number\_43136SRS147039\_contig\_number\_contig-100\_23589.23590SRS148817\_contig\_number\_contig-100\_20098.20099
order

31979
family
12

1485
2
12
genus

SRS050752\_contig\_number\_27887SRS104912\_contig\_number\_3771

411486
species
1

SRS1041038\_contig\_number\_contig-100\_481.100625


SRS045244\_contig\_number\_3205
1
2293018
species

species
59620
1

SRS023583\_contig\_number\_9797


SRS013521\_contig\_number\_14707
1
species
2293022

species
1263064
6

SRS016335\_contig\_number\_44302SRS019693\_contig\_number\_6286SRS1041092\_contig\_number\_648SRS144183\_contig\_number\_38054SRS147377\_contig\_number\_contig-100\_2425.65320SRS893230\_contig\_number\_contig-100\_1623.72970

541000
family
4

1263
3
4
genus

SRS019968\_contig\_number\_16359SRS020233\_contig\_number\_60024SRS050925\_contig\_number\_27468

species
2293182
1

SRS1055043\_contig\_number\_27529

21
186806
family

1730
1
21
genus

SRS015663\_contig\_number\_contig-100\_1304.214279

species
39485

SRS012849\_contig\_number\_36862SRS015190\_contig\_number\_13823SRS019496\_contig\_number\_18183SRS022071\_contig\_number\_39269SRS042284\_contig\_number\_6526SRS043411\_contig\_number\_18156SRS049896\_contig\_number\_34487SRS051031\_contig\_number\_34178SRS064232\_contig\_number\_4277SRS077231\_contig\_number\_26648SRS098881\_contig\_number\_18549SRS1041129\_contig\_number\_12538SRS1041157\_contig\_number\_1014SRS104693\_contig\_number\_10341SRS1055067\_contig\_number\_14194SRS144506\_contig\_number\_57940SRS144537\_contig\_number\_contig-100\_907.251229SRS893358\_contig\_number\_12694SRS893366\_contig\_number\_contig-100\_999.127703SRS893378\_contig\_number\_23967
20

family

SRS011084\_contig\_number\_36947SRS014613\_contig\_number\_7312SRS014683\_contig\_number\_contig-100\_3024.108010SRS014923\_contig\_number\_40247SRS015065\_contig\_number\_6480SRS015264\_contig\_number\_contig-100\_2136.124861SRS015578\_contig\_number\_14780SRS016056\_contig\_number\_contig-100\_13202.13202SRS019178\_contig\_number\_1581SRS020869\_contig\_number\_34238SRS021484\_contig\_number\_32482SRS022524\_contig\_number\_16516SRS023914\_contig\_number\_8303SRS023971\_contig\_number\_725SRS024132\_contig\_number\_5486SRS043001\_contig\_number\_10588SRS045645\_contig\_number\_34792SRS048060\_contig\_number\_12910SRS050026\_contig\_number\_12141SRS052027\_contig\_number\_8275SRS054905\_contig\_number\_13161SRS055982\_contig\_number\_11790SRS057717\_contig\_number\_contig-100\_9172.9172SRS058070\_contig\_number\_16977SRS063040\_contig\_number\_17640SRS064757\_contig\_number\_25376SRS065176\_contig\_number\_contig-100\_1175.109666SRS074964\_contig\_number\_3255SRS075078\_contig\_number\_33275SRS075341\_contig\_number\_24514SRS075963\_contig\_number\_contig-100\_1447.1447SRS077086\_contig\_number\_14765SRS077730\_contig\_number\_18846SRS098571\_contig\_number\_38571SRS101376\_contig\_number\_29598SRS101433\_contig\_number\_contig-100\_972.110597SRS1041033\_contig\_number\_contig-100\_2071.147157SRS1041037\_contig\_number\_17558SRS1041118\_contig\_number\_3375SRS1041132\_contig\_number\_contig-100\_2846.54427SRS1041136\_contig\_number\_27005SRS1041141\_contig\_number\_contig-100\_16420.16421SRS143070\_contig\_number\_18161SRS143181\_contig\_number\_4713SRS144362\_contig\_number\_10198SRS144753\_contig\_number\_contig-100\_18269.63978SRS147272\_contig\_number\_contig-100\_11523.11524SRS147346\_contig\_number\_68552SRS147919\_contig\_number\_17338SRS148159\_contig\_number\_805SRS148253\_contig\_number\_14511SRS148721\_contig\_number\_48683SRS893259\_contig\_number\_5539SRS893285\_contig\_number\_2209SRS893342\_contig\_number\_3752SRS893373\_contig\_number\_contig-100\_635.157006SRS893383\_contig\_number\_19016
186803
57
308

189330
genus
8

39486
species

SRS015579\_contig\_number\_23287SRS023346\_contig\_number\_7623SRS023715\_contig\_number\_22117SRS046717\_contig\_number\_11858SRS053087\_contig\_number\_2624SRS058770\_contig\_number\_28915SRS104084\_contig\_number\_13971SRS142980\_contig\_number\_contig-100\_235.56012
8

1
genus
830

1

SRS024140\_contig\_number\_110
45851
species

6
genus
572511


SRS043667\_contig\_number\_contig-100\_103.45668
1
33039
species


SRS016495\_contig\_number\_13653SRS053214\_contig\_number\_21105SRS1041091\_contig\_number\_22778SRS1041138\_contig\_number\_contig-100\_769.72064SRS143372\_contig\_number\_12189
5
species
33038

2
33042
genus

species
2293096
1

SRS077024\_contig\_number\_15313

1

SRS104636\_contig\_number\_contig-100\_2218.175579
116085
species

species
39491
105

SRS011134\_contig\_number\_33436SRS011405\_contig\_number\_contig-100\_112.115782SRS012969\_contig\_number\_40061SRS013098\_contig\_number\_contig-100\_896.300639SRS013940\_contig\_number\_22634SRS014235\_contig\_number\_contig-100\_2321.258527SRS014459\_contig\_number\_5647SRS014736\_contig\_number\_9362SRS014948\_contig\_number\_24853SRS014979\_contig\_number\_33831SRS015431\_contig\_number\_14059SRS015486\_contig\_number\_contig-100\_331.332SRS015694\_contig\_number\_13183SRS015854\_contig\_number\_24854SRS016203\_contig\_number\_13228SRS016629\_contig\_number\_4049SRS017247\_contig\_number\_10662SRS017433\_contig\_number\_16964SRS017622\_contig\_number\_contig-100\_159.11705SRS017859\_contig\_number\_7917SRS018351\_contig\_number\_20744SRS018817\_contig\_number\_contig-100\_1056.147577SRS018984\_contig\_number\_31492SRS019030\_contig\_number\_contig-100\_10.124506SRS019068\_contig\_number\_19302SRS019286\_contig\_number\_1196SRS019381\_contig\_number\_contig-100\_1319.136242SRS019397\_contig\_number\_9899SRS019445\_contig\_number\_5487SRS019787\_contig\_number\_8504SRS020394\_contig\_number\_12462SRS022609\_contig\_number\_43385SRS024009\_contig\_number\_contig-100\_3.88357SRS024075\_contig\_number\_16792SRS042628\_contig\_number\_35649SRS042966\_contig\_number\_17554SRS045739\_contig\_number\_contig-100\_929.77989SRS045826\_contig\_number\_24763SRS048870\_contig\_number\_22658SRS049712\_contig\_number\_24380SRS049959\_contig\_number\_52509SRS050941\_contig\_number\_20918SRS050998\_contig\_number\_585SRS052697\_contig\_number\_46715SRS053335\_contig\_number\_17880SRS055017\_contig\_number\_contig-100\_1553.209951SRS056273\_contig\_number\_33586SRS063370\_contig\_number\_contig-100\_33.87898SRS063813\_contig\_number\_4853SRS064276\_contig\_number\_41545SRS064557\_contig\_number\_23369SRS064645\_contig\_number\_1377SRS064973\_contig\_number\_contig-100\_3796.3796SRS065397\_contig\_number\_15260SRS074670\_contig\_number\_8999SRS075716\_contig\_number\_4823SRS075821\_contig\_number\_2181SRS075821\_contig\_number\_21262SRS075821\_contig\_number\_contig-100\_4403.4404SRS076976\_contig\_number\_contig-100\_6253.6254SRS077294\_contig\_number\_19249SRS077392\_contig\_number\_38824SRS077641\_contig\_number\_14709SRS078419\_contig\_number\_36971SRS097889\_contig\_number\_6237SRS097958\_contig\_number\_contig-100\_1112.146308SRS098644\_contig\_number\_33745SRS100021\_contig\_number\_12428SRS103987\_contig\_number\_37893SRS1041031\_contig\_number\_31382SRS1041036\_contig\_number\_contig-100\_12145.12146SRS1041145\_contig\_number\_40115SRS1041146\_contig\_number\_contig-100\_591.19070SRS104327\_contig\_number\_547SRS104400\_contig\_number\_53380SRS104485\_contig\_number\_5503SRS105082\_contig\_number\_23449SRS105153\_contig\_number\_30243SRS1055034\_contig\_number\_13388SRS1055056\_contig\_number\_contig-100\_32.82872SRS1055069\_contig\_number\_14376SRS142542\_contig\_number\_26730SRS142712\_contig\_number\_4462SRS142781\_contig\_number\_12187SRS143085\_contig\_number\_6426SRS143466\_contig\_number\_32820SRS143523\_contig\_number\_27444SRS143722\_contig\_number\_contig-100\_1430.90741SRS144603\_contig\_number\_17782SRS144714\_contig\_number\_30319SRS146813\_contig\_number\_27646SRS147271\_contig\_number\_33457SRS147653\_contig\_number\_contig-100\_327.59494SRS148319\_contig\_number\_17866SRS148784\_contig\_number\_8073SRS148874\_contig\_number\_17330SRS148970\_contig\_number\_4505SRS149075\_contig\_number\_19923SRS892882\_contig\_number\_contig-100\_169.83323SRS893231\_contig\_number\_contig-100\_147.49755SRS893252\_contig\_number\_contig-100\_23.117676SRS893327\_contig\_number\_contig-100\_33.76331SRS893341\_contig\_number\_contig-100\_2372.95498SRS893380\_contig\_number\_121SRS971276\_contig\_number\_34402

841
8
118
genus

SRS013476\_contig\_number\_31082SRS018541\_contig\_number\_2411SRS021948\_contig\_number\_37153SRS049773\_contig\_number\_18670SRS057480\_contig\_number\_10503SRS1054691\_contig\_number\_21283SRS893172\_contig\_number\_15742SRS893369\_contig\_number\_12806

species
2293136
1

SRS076756\_contig\_number\_22391

301302
species

SRS013158\_contig\_number\_5494SRS015960\_contig\_number\_23093SRS019161\_contig\_number\_37679SRS056259\_contig\_number\_22280SRS058723\_contig\_number\_8680SRS075878\_contig\_number\_24102SRS077335\_contig\_number\_8904SRS1041032\_contig\_number\_8628SRS143148\_contig\_number\_27104SRS147425\_contig\_number\_contig-100\_114.82144SRS149244\_contig\_number\_13145
11

301301
species

SRS098655\_contig\_number\_7195
1

3

SRS043841\_contig\_number\_8924SRS044535\_contig\_number\_20455SRS046712\_contig\_number\_3430
2049040
species


SRS146888\_contig\_number\_14625
1
1638781
species


SRS1041090\_contig\_number\_8200SRS1055076\_contig\_number\_15799
2
species
2293144

89

SRS011239\_contig\_number\_27996SRS012273\_contig\_number\_contig-100\_554.281498SRS013687\_contig\_number\_24092SRS014412\_contig\_number\_21395SRS014855\_contig\_number\_17920SRS015369\_contig\_number\_933SRS015782\_contig\_number\_28802SRS015890\_contig\_number\_25219SRS016018\_contig\_number\_contig-100\_136.103150SRS016095\_contig\_number\_26955SRS016267\_contig\_number\_8577SRS016954\_contig\_number\_26229SRS017103\_contig\_number\_42590SRS017521\_contig\_number\_35226SRS017916\_contig\_number\_18450SRS018427\_contig\_number\_31800SRS018623\_contig\_number\_11690SRS018656\_contig\_number\_12361SRS019601\_contig\_number\_31366SRS019685\_contig\_number\_43069SRS019808\_contig\_number\_14721SRS023526\_contig\_number\_37664SRS024331\_contig\_number\_38866SRS024492\_contig\_number\_contig-100\_1360.115989SRS024625\_contig\_number\_6769SRS024663\_contig\_number\_4494SRS043701\_contig\_number\_17518SRS043768\_contig\_number\_30109SRS045004\_contig\_number\_33517SRS046068\_contig\_number\_5157SRS046369\_contig\_number\_15834SRS047014\_contig\_number\_36726SRS048164\_contig\_number\_33371SRS049446\_contig\_number\_15650SRS049900\_contig\_number\_contig-100\_3059.124077SRS049995\_contig\_number\_contig-100\_901.222367SRS050299\_contig\_number\_5753SRS051610\_contig\_number\_11039SRS053356\_contig\_number\_47965SRS053398\_contig\_number\_22711SRS053649\_contig\_number\_22643SRS056519\_contig\_number\_contig-100\_1766.107414SRS063190\_contig\_number\_3537SRS074586\_contig\_number\_5313SRS075021\_contig\_number\_8720SRS075773\_contig\_number\_39237SRS075984\_contig\_number\_27951SRS076804\_contig\_number\_20517SRS076929\_contig\_number\_27479SRS077454\_contig\_number\_contig-100\_1721.112593SRS077552\_contig\_number\_9508SRS077849\_contig\_number\_23769SRS078176\_contig\_number\_23755SRS097920\_contig\_number\_30532SRS098061\_contig\_number\_14267SRS098073\_contig\_number\_19335SRS098717\_contig\_number\_14806SRS1041039\_contig\_number\_29648SRS1041130\_contig\_number\_5226SRS1041133\_contig\_number\_27609SRS1041137\_contig\_number\_31644SRS1041139\_contig\_number\_4236SRS1041140\_contig\_number\_23265SRS1041144\_contig\_number\_8762SRS104975\_contig\_number\_25306SRS1055022\_contig\_number\_20418SRS1055049\_contig\_number\_12274SRS1055083\_contig\_number\_13792SRS140492\_contig\_number\_contig-100\_2347.208521SRS142923\_contig\_number\_14549SRS143342\_contig\_number\_20903SRS143780\_contig\_number\_6316SRS143876\_contig\_number\_40815SRS144135\_contig\_number\_18637SRS144297\_contig\_number\_contig-100\_677.107548SRS146764\_contig\_number\_contig-100\_264.206629SRS147139\_contig\_number\_39563SRS147557\_contig\_number\_34569SRS147977\_contig\_number\_10820SRS148196\_contig\_number\_7912SRS148424\_contig\_number\_36330SRS149325\_contig\_number\_contig-100\_1801.111935SRS149784\_contig\_number\_25182SRS149879\_contig\_number\_25816SRS893170\_contig\_number\_contig-100\_293.68186SRS893187\_contig\_number\_20697SRS893295\_contig\_number\_920SRS893300\_contig\_number\_contig-100\_1348.1349SRS971275\_contig\_number\_44382
species
166486

1

SRS143895\_contig\_number\_32858
species
1262945

1

SRS1054928\_contig\_number\_contig-100\_162.50745
360807
species

4
1506553
genus

species
1946694
1

SRS147766\_contig\_number\_14867

208479
species
3

SRS050422\_contig\_number\_37812SRS062654\_contig\_number\_27733SRS148091\_contig\_number\_13899

7
genus
1506577

7

SRS011302\_contig\_number\_29596SRS017701\_contig\_number\_11869SRS018836\_contig\_number\_28016SRS051882\_contig\_number\_5141SRS078242\_contig\_number\_15473SRS145497\_contig\_number\_20725SRS893279\_contig\_number\_17442
29361
species

species
384638
2

SRS024435\_contig\_number\_44011SRS098827\_contig\_number\_13720
